# Acupuncture in Dentistry: A Comprehensive Review of Its Applications, Mechanisms, and Clinical Efficacy

**DOI:** 10.7759/cureus.80246

**Published:** 2025-03-08

**Authors:** Amit Patil, Sayem A Mulla, Waseem Z Khan, Aarti S Bedia, Deepak Sharma, Vyshnavi Mundada

**Affiliations:** 1 Conservative Dentistry and Endodontics, Bharati Vidyapeeth (Deemed to be University) Dental College and Hospital, Navi Mumbai, IND; 2 Dentistry, Bharati Vidyapeeth (Deemed to be University) Dental College and Hospital, Navi Mumbai, IND; 3 Orthodontics and Dentofacial Orthopaedics, Bharati Vidyapeeth (Deemed to be University) Dental College and Hospital, Navi Mumbai, IND; 4 Oral Medicine and Radiology, Bharati Vidyapeeth (Deemed to be University) Dental College and Hospital, Navi Mumbai, IND

**Keywords:** acupuncture, acupuncture therapy, acute pain, dental pain, dentistry, temporomandibular disorder

## Abstract

The ancient therapeutic practice of acupuncture, which has its roots in traditional Chinese medicine, has drawn a lot of interest lately due to its possible use in contemporary dentistry. Examining the mechanics, clinical effectiveness, and contemporary uses of acupuncture in dentistry is the goal of this review. We evaluate its impact on the healing process, surgical recovery, anxiety management, and dental pain. However, more thorough and well-planned randomized controlled trials are still required to confirm its therapeutic advantages. The aim of this review is to explore acupuncture's mechanisms of action, including endorphin release, autonomic nervous system regulation, and effects on local blood circulation, along with how it may be included in a comprehensive dental care plan.

## Introduction and background

Acupuncture has long been recognized in traditional Chinese medicine as a therapeutic technique that stimulates certain body locations to aid in healing and balance restoration. In recent decades, its application has expanded beyond general health and well-being to more specialized fields like dentistry [1]. As an adjunct to conventional treatments, acupuncture has grown in popularity among dentists, particularly for the treatment of pain during and following dental procedures. Due to acupuncture's ability to influence the body's neurological system, generate endorphins, and improve blood circulation, patients undergoing dental operations such as tooth extractions, root canals, and implants may have reduced pain and suffering [2].

Despite acupuncture's growing popularity in dentistry, its effectiveness is still the subject of contradictory scientific research. Numerous studies have attempted to determine its potential benefits, with differing degrees of success. Acupuncture has been shown in certain studies to dramatically reduce pain, anxiety, and the healing process after surgery, providing patients with an alternative to more traditional pain management methods like using medication [3]. However, some studies have not demonstrated a clear, measurable benefit over placebo or traditional pain relief methods. These differences in results have led to ongoing debates over the true efficacy of acupuncture in dental treatment, with critics contending that its benefits are more likely to be the result of placebo or physiological factors [4].

**Figure 1:**
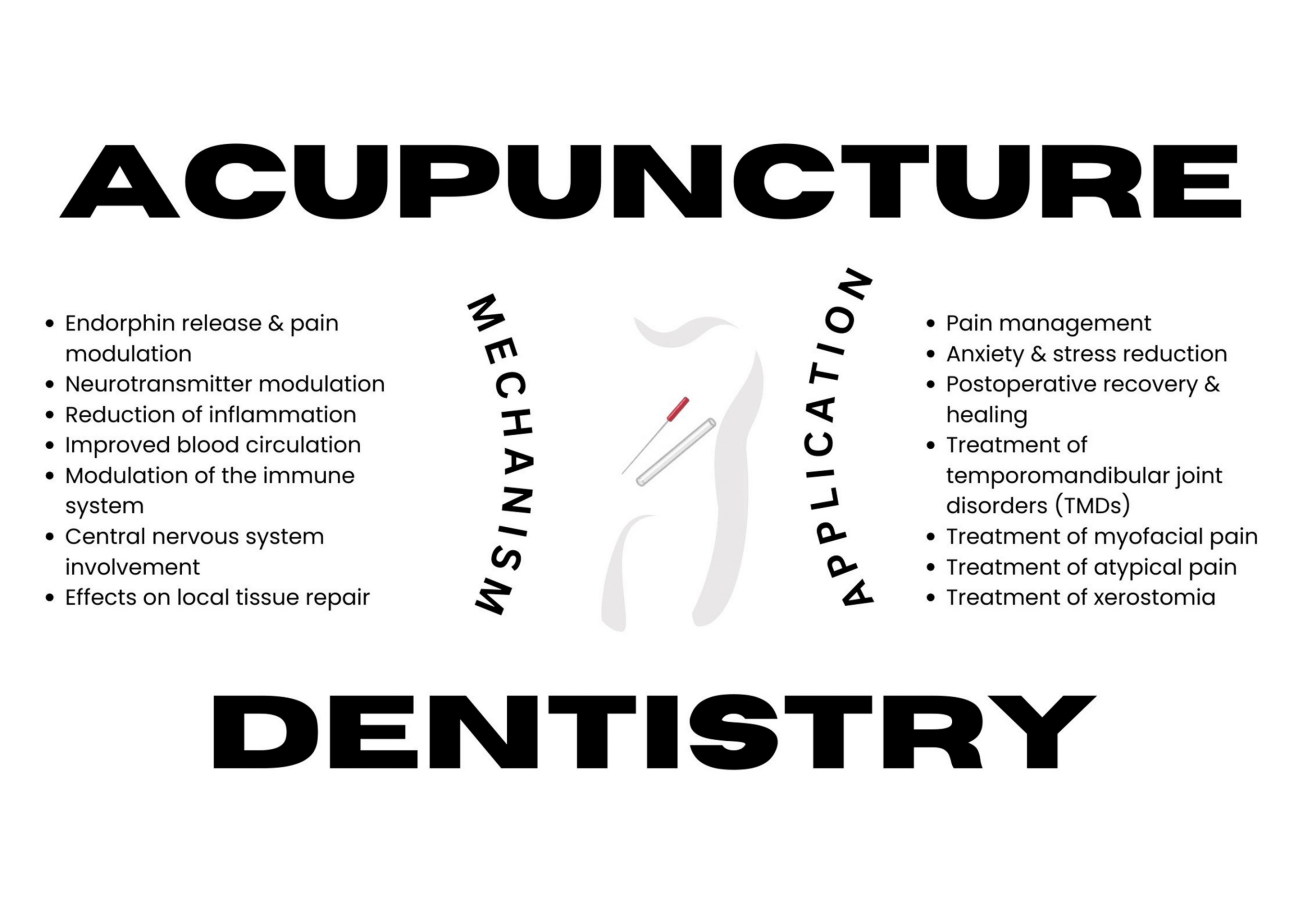
Acupuncture in dentistry: Mechanisms and applications. Image credits: Dr. Sayem A. Mulla.

Because of the contradictory research findings, some members of the dental community still hold the use of acupuncture in dentistry in high regard. Despite the potential advantages, more comprehensive and carefully thought-out clinical research is needed to offer exact application recommendations [5]. Acupuncture may be a helpful tool in dentistry offices if more research validates its benefits, especially when it comes to providing a holistic approach to patient care by reducing the need for prescription medications and fostering a more comfortable and serene treatment atmosphere [6]. Acupuncture's ongoing use in dentistry may depend on improved scientific understanding of its mechanisms and the development of evidence-based practices to support its application (Figure 1). There seems to exist very little literature on the mechanisms by which it acts when dental treatment is considered. Thus, this review aims to highlight the mechanisms involved in the functioning of acupuncture in dentistry while also exploring its multifaceted role in the dental procedure.

Materials and methods

Electronic databases such as the National Library of Medicine (MEDLINE PubMed), Cochrane Library, and Google Scholar were assessed using the key terms "acupuncture" AND "dentistry". All the articles were assessed for possible inclusion wherein acupuncture was used as a treatment modality in dentistry and considered in this review. All English language articles were considered. All the data collected were used in the present review.

Mechanisms of acupuncture in dentistry

It is thought that acupuncture works through a variety of physiological processes. It is believed that a number of important systems are involved in dental operations.

Endorphin Release and Pain Modulation

Acupuncture has long been associated with the natural painkiller endorphins. When acupuncture needles are inserted into certain body sites, they stimulate nerve endings. These nerve fibers, particularly the A-delta and C fibers, provide information to the brain that triggers the production of endorphins and other neurotransmitters. Like synthetic medications, these endogenous opioids significantly reduce the perception of pain by binding to opioid receptors in the brain and spinal cord [7]. As a result, acupuncture can be used to treat dental patients' acute and chronic pain, including pain from issues with the temporomandibular joint (TMJ) and discomfort that occurs after extractions [8].

Moreover, the gate control theory of pain explains how acupuncture regulates pain. According to this theory, acupuncture stimulates larger sensory fibers that "close the gate" in the spinal cord, preventing pain signals from reaching the brain. This kind of natural pain relief can be soothing without the need for prescription drugs [8].

Neurotransmitter Modulation

Acupuncture affects many neurotransmitters, such as serotonin, dopamine, and norepinephrine. Controlling mood, pain, and the body's response to stress all depend on these neurotransmitters. Acupuncture can help patients feel better and suffer less anxiety, which is frequent among individuals having dental treatment done, since it promotes the production of these hormones [9].

For example, serotonin plays a part in mood control and can reduce depression and anxiety, two illnesses that many dental patients encounter. Similarly, the "feel-good" neurotransmitter, dopamine, encourages relaxation by creating a sense of pleasure and well-being. The alteration of these neurotransmitters influences the central nervous system, which reduces anxiety, promotes calmness, and even enhances pain relief [10,11].

Reduction of Inflammation

Acupuncture's anti-inflammatory qualities are believed to be highly beneficial in the dental industry. Needles inserted into specific acupuncture points are thought to enhance the body's immune response and promote the production of anti-inflammatory substances such as cytokines and prostaglandins [12]. This reduces the likelihood that tissue may enlarge and become inflamed after dental surgeries, implants, or extractions [13].

Improved Blood Circulation

Acupuncture may also promote increased blood flow in the affected areas, improving the exchange of waste materials, nutrients, and oxygen between tissues. This can speed up the healing process, reduce swelling, and improve discomfort. For people with inflammatory diseases like periodontitis or TMJ problems, acupuncture may help reduce inflammation and promote healing, perhaps reducing the need for medication [14,15].

Improved circulation in dental patients may reduce postoperative edema and bruises. For example, acupuncture may help speed up the healing of the gums and jawbone after tooth extraction by ensuring that recuperating tissues receive adequate blood flow [16]. Additionally, this faster healing may reduce overall recovery time and discomfort [17].

Modulation of the Immune System

Acupuncture has been shown to influence the immune system by altering the production and operation of signaling molecules and immune cells. When acupuncture needles are inserted into the skin, certain cytokines are produced, which may trigger immunological reactions. By helping to control the body's immunological response, these cytokines promote tissue regeneration and infection prevention [18].

During dental procedures, acupuncture can be very helpful in preventing or reducing the risk of infection. For example, after a tooth extraction, the immune system may need a boost to help fight off any bacterial infections. Acupuncture's ability to control the immune system can also help reduce unnecessary inflammation, which supports a healthy and efficient healing process [19].

Central Nervous System Involvement

Acupuncture has a major effect on the central nervous system (CNS), which includes the brain and spinal cord. By stimulating specific locations on the body, acupuncture activates brain regions involved in processing pain and controlling emotions. Both the cerebral cortex, which interprets pain, and the limbic system, which is involved in emotions and stress responses, are impacted by acupuncture [20].

Acupuncture also has an impact on the hypothalamic-pituitary-adrenal (HPA) axis, which controls the body's response to stress. Because acupuncture balances the autonomic nervous system, it can help patients feel more at ease and relaxed throughout dental operations. This makes it especially beneficial for people who have dental anxiety or stress related to dental procedures [21]. By calming the nervous system, acupuncture improves the general comfort and enjoyment of dental care.

Effects on Local Tissue Repair

Acupuncture may promote local tissue repair by promoting the synthesis of collagen and growth factors, two essential components of wound healing. The body needs to regenerate and repair damaged tissues after dental treatments like root canals, implant surgeries, or tooth extractions [22]. The potential benefits of acupuncture include enhancing the body's ability to repair itself.

Stimulating acupuncture sites has been shown to improve the healing process overall, reduce scarring, and encourage tissue regeneration [23]. The ability of acupuncture to stimulate growth factors can enhance tissue regeneration during dental procedures that require bone or soft tissue repair, leading to shorter recovery times and improved outcomes.

Psychological and Emotional Benefits

Acupuncture offers psychological and emotional benefits in addition to its physical therapeutic effects. Dental patients frequently experience significant anxiety, fear, or tension, particularly before or during a procedure. Acupuncture can help reduce these feelings by enhancing the autonomic nervous system and promoting a parasympathetic (rest and digest) response, which counteracts the stress-related sympathetic (fight-or-flight) system [24].

By encouraging relaxation, acupuncture can make patients feel more comfortable and calmer during dental visits. It has been shown to provide emotional support and reduce anxiety, improving patients' overall comfort and experience [25]. The placebo effect, which happens when patients believe the treatment is working, can significantly intensify the psychological benefits of acupuncture [26].

Specific Applications in Dentistry

Acupuncture has several specific applications in dentistry that might benefit patients undergoing various dental operations. It is commonly used to relieve pain in conditions including toothaches, TMJ issues, and postoperative rehabilitation. Acupuncture may be particularly helpful in controlling pain after teeth extractions or surgeries because it helps reduce inflammation, releases chemicals that decrease pain, and promotes circulation to aid in healing [27].

In addition to treating pain, acupuncture is commonly used to lessen anxiety. It has been shown that acupuncture helps patients feel more at rest by reducing their fear or anxiety before dental procedures. Another advantage of acupuncture is its ability to control nausea, particularly for those who may have this side effect after sedation or anesthesia. Myofascial pain syndrome and bruxism, conditions that result in pain in the muscles surrounding the jaw, may also benefit from acupuncture [28].

## Review

Applications of acupuncture in dentistry

Pain Management

Acupuncture has gained popularity in dentistry due to its ability to effectively treat a wide range of ailments, particularly those related to dental procedures. Preoperative and postoperative pain are among the main scenarios in which acupuncture has shown promise in easing discomfort. Through the stimulation of certain locations along the body's meridian pathways, acupuncture can improve blood circulation and aid in the release of endorphins [29]. This can ease pain and lessen inflammation. Studies suggest that by lowering the need for pharmacological treatments like opioids, acupuncture may help patients manage their pain more comprehensively and perhaps safely before and after dental operations. Furthermore, acupuncture's capacity to promote relaxation can help reduce stress and anxiety, two elements that are commonly linked to heightened pain perception in dental settings [30].

Acupuncture is an alternate or supplemental treatment for long-term dental issues such as temporomandibular joint dysfunction (TMD) and the pain experienced after orthodontic procedures or tooth extractions. By focusing on points that reduce muscle tension and enhance joint function, acupuncture can help treat TMD, which frequently results in jaw discomfort and restricted movement. Similarly, by activating particular acupuncture sites that control the body's pain response systems, acupuncture has been shown to alleviate post-extraction discomfort and decrease edema [31,32]. Furthermore, some research indicates that by relaxing the muscles surrounding the teeth and jaws, acupuncture may lessen the severity of discomfort related to orthodontic therapy, including pain from brace adjustments. All things considered, acupuncture is a useful technique in contemporary dental treatment due to its adaptability in treating both acute and chronic tooth pain [33].

Anxiety and Stress Reduction

Dental anxiety is a common condition that can keep people from getting the care they need, which can result in a cycle of untreated dental issues and increased anxiety at every appointment. Both the patient and the dental team may find treatment more difficult as a result of this anxiety, which can intensify the patient's impression of pain and suffering during operations [34]. By affecting the autonomic nerve system (ANS), which regulates the body's automatic processes, including digestion, stress reaction, and heart rate, acupuncture offers a non-invasive, drug-free remedy for this issue. The parasympathetic (rest and digest) and sympathetic (fight or flight) nervous systems are both triggered by stimulating particular acupuncture sites. This lessens the elevated tension and worry that many patients feel both before and during dental appointments [35].

Furthermore, acupuncture's capacity to stimulate the production of serotonin and endorphins is essential for treating dental anxiety. Serotonin is a neurotransmitter that helps control mood and anxiety, while endorphins are natural pain relievers that encourage emotions of relaxation and well-being. Acupuncture can help patients feel more relaxed during dental treatments by raising the amounts of these chemicals in the brain, which can lessen the need for sedatives or anxiolytic drugs [33]. Because of this, acupuncture is a desirable substitute for people who are hesitant or unable to employ pharmacological therapies because of sensitivities, allergies, or personal preferences. Acupuncture, a non-pharmacological method, can effectively reduce anxiety and provide a more pleasant and comfortable dental visit.

Postoperative Recovery and Healing

The efficacy of acupuncture to facilitate a quicker recovery following dental operations, including extractions, implant placements, and periodontal treatments, is becoming more widely acknowledged. To improve local circulation and strengthen the body's inherent healing processes, the therapy stimulates particular acupuncture sites. Acupuncture helps provide vital nutrients and oxygen to tissues by increasing blood flow to the damaged region, which can hasten the healing process [36]. Additionally, by promoting the removal of extra fluid and reducing inflammation, acupuncture might lessen edema or postoperative swelling. Patients experience less discomfort and recover more quickly as a result of these combined benefits, which improve the healing environment [22].

Additionally, acupuncture significantly lowers the risk of postoperative problems by regulating the body's inflammatory response. Although severe or protracted inflammation can impede recovery and raise the risk of infection or delayed healing, inflammation is a normal component of the healing process. This inflammatory reaction can be controlled by acupuncture to keep it within the ideal range for recovery [19]. Acupuncture is a useful supplement to conventional postoperative therapy by lowering pain and inflammation and promoting tissue regeneration. This may lessen the need for further pain medication and antibiotics and enable patients to resume their regular activities sooner [14].

Treatment of Temporomandibular Joint Disorders

A prevalent cause of orofacial pain is temporomandibular joint disorder, which frequently manifests as joint dysfunction, muscular strain, and inflammation in the jaw region. The disorder may show up as headaches, trouble opening the mouth, or discomfort in the jaw, face, or neck. Because it can address the underlying causes that contribute to TMD, acupuncture has become a viable therapeutic option [27]. Acupuncture can reduce inflammation in the afflicted joints, relax the muscles that might be causing tension or discomfort, and promote the body's natural healing processes by focusing on particular acupuncture points. This all-natural method of treating TMD gives patients a drug-free substitute that may either supplement or, in certain situations, take the place of more conventional therapies like physical therapy or painkillers [28].

Acupuncture's efficacy in reducing TMD symptoms has been substantiated by several clinical investigations, which have demonstrated notable enhancements in jaw movement, muscle relaxation, and pain reduction. In addition to relieving pain by encouraging the production of endorphins, acupuncture's effects on muscular relaxation provide improved mobility and less jaw stiffness [31]. Acupuncture has also been demonstrated to increase blood flow to the afflicted location, which promotes the repair of inflammatory tissues and lessens the general pain brought on by TMD. All things considered, acupuncture provides a thorough and efficient therapeutic approach that addresses the joint and muscle components of TMD, assisting patients in regaining their mobility from this frequently incapacitating ailment [32].

Treatment of Myofascial Pain

Localized, hypersensitive areas in palpably tense bands of muscle fibers (myofascial trigger points) are the hallmark of myofascial pain. These trigger points can be brought on by trauma-induced muscular overload or repetitive motions that put certain muscle groups under unusual tension. Clinically, patients report headaches, muscular weakness, stiffness, and limited jaw mobility [37]. Park et al. [38] used a more focused strategy. A total of 27 participants with TMD myofascial discomfort were examined by these researchers using either sham or verum acupuncture at two acupoints over the course of six treatment sessions. They used the Park Sham Device. Verum acupuncture significantly reduced myofascial pain symptoms and indicators, whereas sham acupuncture did not, according to the study. One of the study's main limitations was the low baseline pain levels of the sham acupuncture group participants (average visual analog scale (VAS) score of 1.3), which limited the potential for improvement in pain levels following treatment.

Shen et al. [39] conducted randomized clinical research to assess the efficacy of acupuncture for jaw muscle myofascial discomfort. Verum or sham acupuncture was randomly assigned to 28 participants over the age of 18 who had been diagnosed with persistent myofascial discomfort of the jaw muscles. A numerical rating scale was used to collect assessments of general head and neck discomfort both before and after therapy. Before and after treatment, a mechanical pain stimulus was applied to the masseter muscle, and the degree of pain tolerance was assessed using a VAS. Verum acupuncture significantly decreased subjects' jaw discomfort, jaw/face stiffness, and neck pain while also significantly increasing their masseter muscle's pain tolerance. The subjects were unable to distinguish between sham and verum acupuncture. The group that received sham acupuncture did not see any appreciable decreases in pain.

Treatment of Atypical Pain

Patients who did not respond "typically" to neurosurgical treatments were initially referred to as having atypical face discomfort. Although no precise diagnostic standards have ever been established, the word has been used to refer to a variety of facial discomfort issues and has been thought to indicate a psychological illness. It is well accepted that acupuncture stimulates the nervous system and releases certain messenger molecules that are neurochemicals. These biochemical alterations impact the body's homeostatic processes, hence enhancing patients' mental and physical health. It has been demonstrated that stimulating specific acupuncture sites can influence brain regions that are known to lessen sensitivity to stress and pain [37,40].

Clinical evidence and efficacy

Acupuncture's use in dentistry is supported by a diverse body of clinical evidence, including both promising and concerning results. Acupuncture has been demonstrated in several trials and systematic reviews to be useful in treating both acute and chronic dental pain, lowering anxiety, and enhancing recovery following surgery. Numerous studies, however, have methodological flaws, such as small sample populations, inconsistent acupuncture regimens, and brief follow-up times [41].

Acupuncture dramatically decreased the level of pain experienced by patients having tooth extractions, according to a meta-analysis of the practice's use in dental pain treatment. Acupuncture has also been demonstrated to be successful in lowering the need for analgesics after invasive dental procedures. Notwithstanding these encouraging results, additional thorough, extensive randomized controlled trials (RCTs) are still required to verify its effectiveness and determine the ideal therapy parameters (such as needle insertion sites, length, and frequency).

Treatment of Xerostomia

One of the complementary treatments for xerostomia that has been utilized since the 1980s in Western medicine is acupuncture. It may help patients who do not respond to pilocarpine after receiving radiation therapy for head and neck cancers [42,43]. There are several potential theories regarding its mechanism, which are: neuropeptides released during acupuncture therapy have anti-inflammatory effects on blood flow and trophic advantages on the salivary gland acini, the neural circuit, which activates the salivary nuclei in the pons and, subsequently, the salivary glands via the cranial nerves, may be accessed by acupuncture therapy and neural activations seen when salivary production is increased when the parasympathetic nerves are stimulated.

Treatment of Neural Disorders

Acupuncture can be utilized to address several neurological conditions, such as postherpetic neuralgias, numbness or loss of sensation in the lower lip after the extraction of lower third molars, facial nerve paralysis, and various types of neuralgia, including trigeminal [44]. The application of acupuncture for Bell's palsy is rooted in traditional Chinese medicine (TCM), which suggests that by manipulating needles at both local and distant sites, the flow of Qi in the meridians can be balanced, Qi and blood stability can be restored, and the body's defenses against ailments caused by external wind can be strengthened. Additionally, acupuncture may contribute to enhancing neuronal excitability and promoting nerve fiber regeneration [45].

Certain studies have suggested a connection between acupuncture and the autonomic nervous system. Based on TCM, acupuncture is believed to restore the equilibrium of Yin and Yang, a concept that may align with the contemporary understanding of acupuncture as "acupuncture regulates the imbalance between parasympathetic and sympathetic activity" [46]. Local acupuncture points for facial palsy can be found near the masseter muscle prominence, which is situated close to the angle of the mandible, as well as in the indentation between the zygomatic arch and the mandibular notch. These two sites are located in proximity to the branches of the facial nerve [47].

In Silva's study [44], a group of 42 patients with trigeminal neuralgia underwent a 10-day regimen of low-frequency electroacupuncture sessions each day. The treatments were administered in three separate sessions, with a one-week interval in between each. Out of the participants, 36 experienced complete relief, four had partial relief, and two encountered a lack of effectiveness from the therapy.

Challenges and limitations

Despite the encouraging potential advantages of acupuncture in dentistry, there are a number of obstacles and restrictions preventing its widespread use. First off, the interpretation of findings is made more difficult by the variation in acupuncture procedures, including the particular acupuncture sites employed and treatment regimens. Furthermore, the effectiveness of acupuncture may be limited due to the absence of standardized training for dental practitioners who want to use it in their practice. Furthermore, in acupuncture trials, the placebo effect is still an issue. Even while acupuncture has physiological effects, it can be challenging to distinguish between the psychological impacts of therapy and the actual therapeutic advantages. To reduce bias and produce more trustworthy results, blinding and sham-controlled experiments are crucial.

## Conclusions

With potential advantages in pain management, anxiety reduction, and postoperative healing, acupuncture offers a promising supplement to conventional dentistry therapies. Although early data points to promising results, there is not enough high-caliber, extensive research to make acupuncture a firm recommendation as a standard component of dental care. Its effectiveness, ideal application procedures, and long-term advantages require more investigation, especially well-designed RCTs. Acupuncture may prove to be a useful treatment for dental disorders with more research, improving patient care holistically.
